# A Dosimetric Parameter Reference Look-Up Table for GRID Collimator-Based Spatially Fractionated Radiation Therapy

**DOI:** 10.3390/cancers14041037

**Published:** 2022-02-18

**Authors:** Hualin Zhang, Michael P. Grams, Joseph J. Foy, Nina A. Mayr

**Affiliations:** 1 Department of Radiation Oncology, Northwestern University Feinberg School of Medicine, Northwestern University, Chicago, IL 60611, USA; 2 Department of Radiation Oncology, Northwestern Memorial Hospital, Chicago, IL 60611, USA; joseph.foy@nm.org; 3 Department of Radiation Oncology, Mayo Clinic, Rochester, MN 55905, USA; grams.michael@mayo.edu; 4 Department of Radiation Oncology, University of Washington School of Medicine, Seattle, WA 98195, USA; ninamayr@uw.edu; 5 Tumor Heterogeneity Imaging and Radiomics Laboratory, University of Washington School of Medicine, Seattle, WA 98195, USA

**Keywords:** GRID therapy, spatially fractionated radiation therapy, equivalent uniform dose, peak valley dose ratio, reference table

## Abstract

**Simple Summary:**

Dose prescription for the inhomogeneous dosing in spatially fractionated radiation therapy (SFRT) is challenging, and further hampered by the inability of several planning systems to incorporate complex SFRT dose patterns. We developed dosing reference tables for an inventory of tumour scenarios and tested their accuracy with water phantom measurements of GRID therapy, delivered by a standard commercial GRID collimator. We find that dose heterogeneity parameters and *EUD* modeling are consistent across tumour sizes, configurations, and treatment depths. These results suggest that the developed reference tables can be used as a practical clinical resource for clinical decision-making on GRID therapy and to facilitate heterogeneity dose estimates in clinical patients when this commercially available GRID device is used.

**Abstract:**

Computations of heterogeneity dose parameters in GRID therapy remain challenging in many treatment planning systems (TPS). To address this difficulty, we developed reference dose tables for a standard GRID collimator and validate their accuracy. The .decimal Inc. GRID collimator was implemented within the Eclipse TPS. The accuracy of the dose calculation was confirmed in the commissioning process. Representative sets of simulated ellipsoidal tumours ranging from 6–20 cm in diameter at a 3-cm depth; 16-cm ellipsoidal tumours at 3, 6, and 10 cm in depth were studied. All were treated with 6MV photons to a 20 Gy prescription dose at the tumour center. From these, the GRID therapy dosimetric parameters (previously recommended by the Radiosurgery Society white paper) were derived. Differences in D5 through D95 and *EUD* between different tumour sizes at the same depth were within 5% of the prescription dose. *PVDR* from profile measurements at the tumour center differed from D10/D90, but D10/D90 variations for the same tumour depths were within 11%. Three approximation equations were developed for calculating *EUD*s of different prescription doses for three radiosensitivity levels for 3-cm deep tumours. Dosimetric parameters were consistent and predictable across tumour sizes and depths. Our study results support the use of the developed tables as a reference tool for GRID therapy.

## 1. Introduction

Spatially fractionated radiation therapy (SFRT) is a specially designed radiation therapy modality characterized by the delivery of a high and intentionally heterogeneous dose using megavoltage x-ray or proton beams for the management of patients with bulky tumours. Photon beam GRID therapy commonly employs commercially available GRID block collimators or multi-leaf collimator (MLC) modulation to generate the heterogeneous dose pattern by converting an open photon treatment field into multiple small beamlets, which are collimated into a well-defined pattern.

An ample body of literature has shown drastic and often rapid tumour responses to SFRT, with unexpectedly low toxicity rates in the treatment of bulky, recurrent, or therapy-refractory tumours [1,2,3,4,5,6].

Most of the clinical SFRT experience is derived from GRID block collimator (here referred to simply as GRID collimator, or GRID) techniques. While a complete understanding of the radiobiological underpinnings of the observed SFRT responses is still an area of continued investigation, both shallow and deep-seated bulky tumours have consistently shown high symptomatic response rates to collimator-based GRID therapy [1,2,5,7,8,9,10,11,12,13,14,15,16]. These encouraging clinical outcomes of SFRT were seen initially in palliative therapy, showing response rates in excess of 90% in patients with bulky refractory metastases and recurrences from various primary tumours [1]. More recently, the GRID experience has expanded increasingly into the treatment of far-advanced primary tumours, particularly advanced non-metastatic head and neck cancer, lung cancer, and sarcoma, which demonstrate higher than expected long-term local control outcomes, low toxicity, and encouraging survival outcomes [1,2,5,12,13,14,15,16]. In particular, a large nominal dose of SFRT did not reduce the patients’ radiation tolerance even when followed by full-dose conventional radiation therapy [5,14]. These clinical outcomes suggest that hypofractionated GRID therapy can be safely incorporated into the definitive management of both bulky recurrent/metastatic and likely far-advanced primary tumours. These promising pilot results, in conjunction with the continued technical advances in radiotherapy dose modeling and delivery technologies, have spawned an accelerated interest in SFRT and growth in newly established centers aiming to initiate SFRT as a treatment modality for their patients.

GRID therapy is generally delivered with commercially available GRID block collimators. While SFRT techniques other than GRID, such as Lattice (a 3D form of GRID therapy), have been developed, due to the fixed geometric properties, dosimetric consistency, and convenience of straightforward delivery, GRID collimator-based SFRT treatment continues to be widely practiced and prevalent in most treatment centers per an RSS GRID/Lattice, Microbeam, and FLASH Working Groups survey. With these properties, GRID collimators are an attractive platform for both high-volume practices and for those lacking access to highly advanced technologies, as well as an entry technology for institutions that are starting new SFRT programs.

However, for clinical physicists and radiation oncologists, the profound departure of SFRT from the well-established dosimetric and planning principles poses new and unfamiliar challenges. In view of the inherent complexity associated with the heterogeneous dose patterns in SFRT and the potential difficulty in adapting current treatment planning systems (TPS) to GRID dosimetry, there is a need to further facilitate dosimetric computations and treatment planning while remaining clinically effort- and time-efficient, particularly for entry-level centers that seek to establish a GRID program.

Dosimetric reference tables have been widely employed in radiation oncology for treatment planning, for example, the prostate seed implant nomograms and eye-plaque seed activity look-up tables. We have adapted this overall concept to the use of GRID collimator-based SFRT. Collimator-based GRID therapy lends itself very well to the concept of reference tables because commercially available GRID collimators are generalized devices with standard physical and geometric parameters. We therefore hypothesize that reference tables of dosimetric parameters generated from an array of representative treatment delivery scenarios can provide a robust and validated representation of the complex GRID dosimetry and may thereby be useful as a tool for practical clinical application in GRID therapy.

The purpose of this study was to develop and validate practically applicable GRID dosimetry reference tables for use by physicists, dosimetrists, and radiation oncologists for formulating GRID therapy treatment plans. Specifically, we studied the consistency of the developed reference tables across different tumour geometry, depth, and field size characteristics. The accuracy of a TPS for calculating GRID therapy dose distributions was verified by experimental testing first. The dose–volume histograms (DVH) of sample tumours were used to derive SFRT heterogeneity and modeling metrics; subsequently, the reference tables were generated.

## 2. Materials and Methods

The reference tables were developed and tested based on a commercially available GRID collimator. The Eclipse planning system (Eclipse V15.6, Varian Oncology System, Palo Alto, CA, USA) was used. Tables were derived for the array of tumour depths, sizes, and geometries from the established planning system Eclipse, and subsequently calculated for their peak/valley dose ratio (*PVDR*), the doses covering 95%, 90%, …, 10%, and 5% of tumour volume (D95, D90, …, D10, D5) and equivalent uniform dose (*EUD*).

### 2.1. GRID Collimator: Rationale and Description

**Figure 1:**
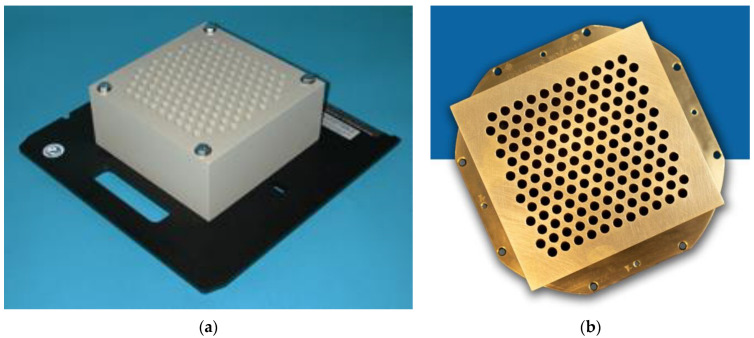
(**a**) Commercially available GRID collimator by High Dose Radiation Grid, Radiation Products Design, Albertville, MN, USA. The image is provided by the vendor. (**b**) Commercially available GRID collimator by .decimal Inc.^TM^, Sanford, FL, USA. The image is provided by the vendor.

Two GRID collimators are currently commercially available, the High Dose Radiation GRID^TM^ collimator (Radiation Products Design, Albertville, MN) (Figure 1a), and the .decimal Inc.^TM^ collimator (Sanford, FL, USA) (Figure 1b). Because of different materials (Cerrobend vs. brass) and different hole sizes, dose distributions and the peak/valley dose ratios at any given depth differ between the two GRID collimators. As an initial demonstration project, this study focuses on the .decimal Inc.^TM^ GRID collimator, one of two commonly used platforms.

**Figure 2:**
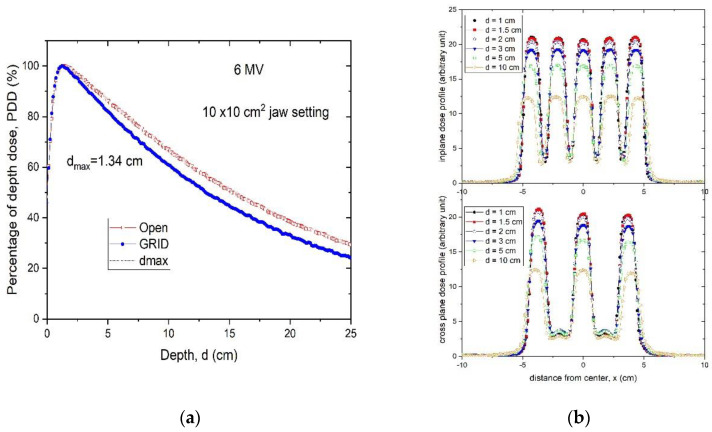
(**a**) Percent depth-dose curves of a 10 × 10 cm^2^ open field and a 10 × 10 cm^2^ GRID collimated field. (**b**) Radial and transverse dose profiles of GRID therapy measured in a water tank at 1, 1.5, 2, 3, 5 and 10 cm depths for a 6 MV beam for the brass .decimal Inc.^TM^ GRID collimator.

The .decimal Inc.^TM^ GRID collimator is an 18 kg brass collimator. The thickness of the brass block is 7.5 cm. The diameter of the holes is 0.80 cm on the upstream side and 1.00 cm on the downstream side, and the center-to-center separation of the holes on the block is 1.40 cm on the lower surface. With this design, approximately 50% of the tissue in the collimated areas is irradiated by the collimated primary beam; the remainder is shielded by the brass block. All holes in the GRID are divergent. Dosimetric characteristics of the .decimal Inc.^TM^ are shown in Figure 2a,b.

The specifications of the .decimal Inc. collimator differ from that of the original GRID collimator by Radiation Products Design and consist of a 7.5 cm thick Cerrobend block with a hexagonal pattern (Figure 1a,b) of circular divergent holes, designed to be mounted in the standard linear accelerator accessory mount. The diameter of the holes is 0.60 cm on the top side and 0.85 cm on the lower side, and the center-to-center separation of the holes on the block is 1.15 cm on the lower surface.

A Blue Phantom^TM^ (Blue Phantom 2, IBA Dosimetry GmbH, Germany) water scanning system was used to perform in-water scans. The profiles were taken using a PTW (Freiburg, Germany) microDiamond Type 60019 detector having a sensitive volume of 0.004 mm^3^. Water tank measurements indicate that, although the percent depth dose (PDD) curve has noticeably changed compared to the open field, the depth of dose maximum (d*max*) remains the same for this hexagonal pattern GRID design. Thus, when we generate plans and perform quality assurance (QA) measurements, we can still use the d*max* depth measured from the open field (Figure 2a), which is a significant convenience.

Figure 2b illustrates the characteristic peak and valley dose distribution of the GRID field for the .decimal Inc.^TM^ GRID collimator. The peak-to-peak distances are different along the radial and transverse directions due to the honeycomb-like hole pattern (Figure 1a). The peaks and valleys are spatially correlated with the collimator apertures, which produce the spatially fractionated dose distribution. Because of manufacturing accuracy limitations small variations in the peaks between different holes can be seen.

From the scanned dose profiles at different depths, the peak/valley (some studies report the valley/peak ratio, VPDR) dose ratio (*PVDR*) can be defined as follows:

(1) $$PVDR=\frac{D_{peak}}{D_{valley}}$$

**Figure 3:**
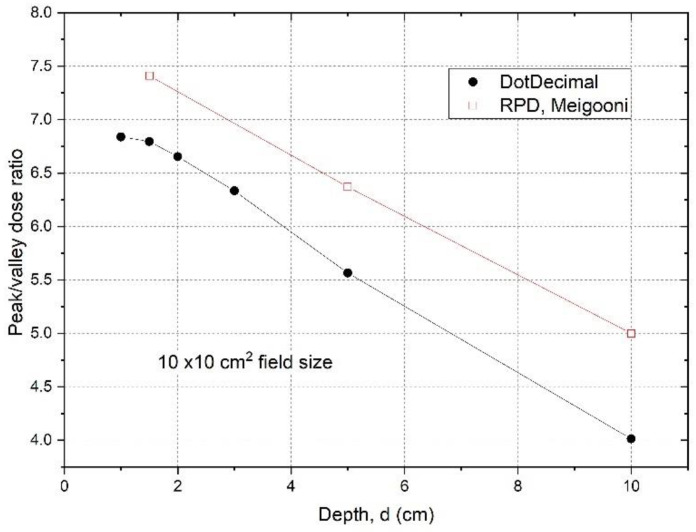
Peak/valley dose ratios were determined from measured dose profiles at different depths using a 10 × 10 cm^2^ field size for the .decimal Inc.^TM^ GRID collimator. The data for a Cerrobend GRID collimator made by Radiation Protection Designs^TM^ (RPD), measured by Meigooni et al. [17] were plotted for comparison. The peak and valley doses are from or near the central axis. The off-axis values are the same (within 2%), because the apertures were designed to be divergent and to deliver the same dose from different holes.

*D_peak_* and *D_valley_* represent the peak and valley doses at a certain depth d. When we determined the peak dose, we averaged the dose over 4 mm at the peak, ±2 mm around the center of peak. For valley dose, we searched the valley of dose profiles, and averaged the dose over ±2 mm distance around the valley minimum dose. Figure 3 shows the *PVDR* of the .decimal Inc.^TM^ GRID collimator determined from the dose inline profiles taken from water tank scans as a function of depth.

The *PVDR*s are variable and dependent on depth if the GRID collimator configuration and beam energy are fixed. For the .decimal Inc.^TM^ GRID collimator with a 6MV beam, the *PVDR* ranged from 6.8 to 4.0 (or the VPDR ranged from 0.15 to 0.25), when the depth was changed from 1 cm to 10 cm. For the Radiation Products Designs^TM^ Cerrobend GRID collimator using a 6 MV beam, it was reported that when depth was increased from 1.5 cm to 10 cm, the *PVDR* decreased from 7.4 to 5.0 (or VPDR increased from 0.14 to 0.20) [17].

### 2.2. Planning Approaches with a GRID Collimator

(a). GRID therapy planning without a TPS

To develop and test the reference tables, we simulated a scenario where the GRID collimator could not be implemented within the TPS for dose calculation. In this case the monitor units (*MU*) needed for delivering the prescribed peak dose (i.e., 15 or 20 Gy) are calculated based on the output factor of the central hole, usually near or passing through the beam’s central axis. The approach is the same for multileaf collimator formed GRID fields, in which an experimental measurement is involved to determine the output factor for delivering the prescribed peak dose at the tumour center depth.

In a patient treatment, the jaws and/or multileaf collimator (MLC) can be used to reduce the field size to create a conformal field adapted to the tumour size. Because the output factor of the GRID field central hole at d*max* (*OUT*_d*max*_) will vary with the field size it needs to be measured. If the dose *D_p_* is prescribed at the depth of d*max*, the *MU* is calculated according to the following:

(2) $$MU=\frac{D_{p\left(Gy\right)}}{OUT_{dmax}\;\left(\frac{Gy}{MU}\right)}$$

However, the depth of the tumour center may be variable, and the treating physician may prescribe the dose to a depth (*d*) other than d*max*. In addition, if two opposed GRID fields are used [18] then the combined PDD curves would be entirely different. If this is the case, the GRID field output factor must be measured at the depth *d*. If the output factor of the GRID field at the depth d is *OUT_d_*_,_ then,

(3) $$MU=\frac{D_{p\left(Gy\right)}}{OUT_{d}\;\left(\frac{Gy}{MU}\right)}$$

It should be noted that because of the dosimetric characteristics of megavoltage X-ray beams, if the dose prescription depth is beyond d*max* the maximum dose delivered will be greater than the prescription dose (the deeper the prescription depth, the greater the maximum dose delivered).

(b). GRID therapy planning using a TPS

To develop the reference tables, we generated plans for different tumour sizes, shapes (either spherical or ellipsoidal) and depths in a flat phantom. The Eclipse system is one of the few TPS that can perform dose computations for commercial GRID collimators. In order to do this, a DICOM file containing the specific geometric features of the GRID collimator, generated by the vendor, had been installed earlier into our TPS per the vendor’s specific instructions.

**Figure 4:**
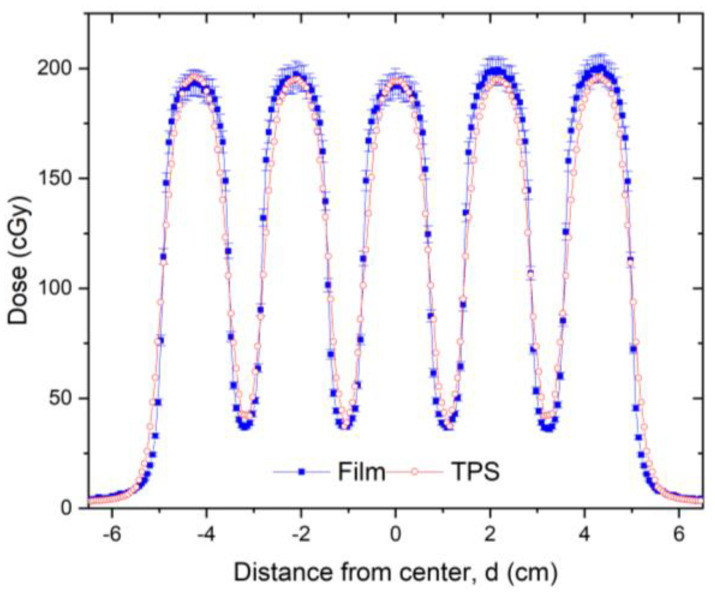
Dose profile comparison between TPS (red line) and radiochromic film measurement (blue line) for a 10 × 10 cm^2^ field made at 5 cm depth in solid water for a 6 MV beam. Radiochromic film measurements can be made for additional checks of TPS calculations and *MU* verification. The 3% error bars were added to the film data. In this figure, a transverse profile comparison was used as an example. In this dose validation process, a gamma analysis was performed resulting in a 99% passing rate with a 3% (global)/3 mm criterion using a 10% (global) minimum dose threshold.

Following installation of the GRID collimator in the TPS, its calculation accuracy was verified. For this verification, water tank scans of the percentage depth dose curves and dose profiles were performed at different depths for various field settings and compared with the TPS-generated counterparts. A calibrated ion-chamber suitable for small field dosimetry or film was used to verify the absolute dose delivered via a GRID field, and a ≤3% difference, which is generally considered acceptable for radiation therapy plan point dose measurement, was set as a tolerance limit. The radiochromic film was calibrated and its accuracy was verified prior to use using a previously published protocol [19]. The software and protocol used was FilmQA Pro (Ashland, Wilmington, DE, USA) and is described in the following reference [20]. Figure 4 presents a dose profile comparison between the TPS and film measurement for a 10 × 10 cm^2^ field size. After the GRID dose calculation accuracy is confirmed, the GRID collimator can be used in the TPS for creating patient treatment plans. Similar to intensity modulated radiation therapy (IMRT), a patient-specific treatment plan QA can be performed to verify the delivered dose.

(c). Tumour size and geometry selection for generating reference tables

**Figure 5:**
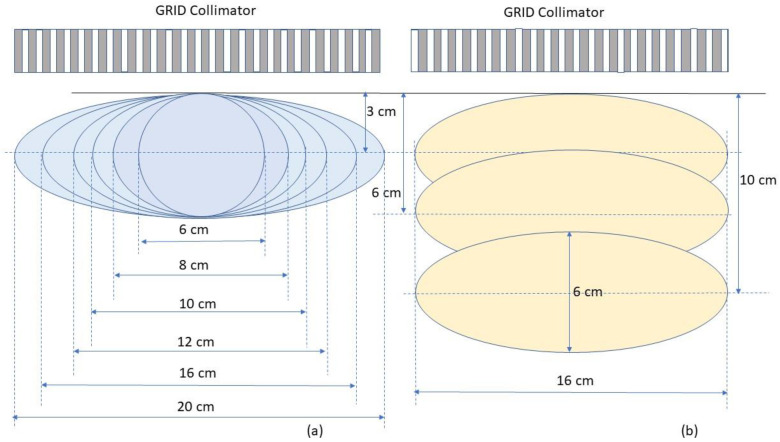
Schematic diagram of tumor geometry and depths used in the test plans. (**a**) shows ellipsoidal tumours with the same height of 6 cm but different sizes in the transverse dimension located at the depth of 3 cm. (**b**) shows tumours with a height of 6 cm and transverse dimension of 16 cm located at the depths of 3, 6 and 10 cm, respectively.

To determine the dosimetric impact of tumour sizes and depths, we chose ellipsoidal tumours ranging from 6. to 20 cm in diameter located either at 3 cm, 6 cm or 10 cm depth (Figure 5). These geometries were selected to generalize the tumour sizes and locations to represent those commonly seen in clinical patients with bulky tumours. Among all tumours investigated, the 6 cm tumour was spherical and other tumours were ellipsoids. The ellipsoidal tumours’ longest diameters were 8, 10, 12, 16 and 20 cm in a plane perpendicular to the beam axis located at either 3, or 6 or 10 cm depth, the tumour height (along the beam axis) was 6 cm. In a separate test, a 20 cm diameter ellipsoidal tumour with 16 cm height located at 8 cm depth was used to verify the reference table. In all tested cases, the dose prescription point was at the center of the tumour volume. A prescription dose of 20 Gy at the 100% isodose line was used.

**Figure 6:**
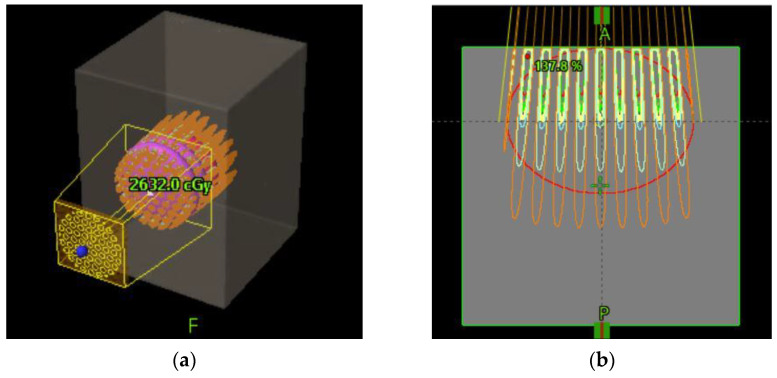
Schematic diagram of GRID collimator (**a**) and dose projection (**b**) in an ellipsoid shaped tumor shown in a TPS.

Figure 6 schematically shows how a GRID collimator is used in a TPS and dose projections from the GRID apertures.

(d). Equivalent uniform dose (*EUD*) calculation

By applying the modified linear quadratic (MLQ) model, we calculated the average surviving fraction, and then derived the corresponding *EUD* from a GRID therapy dose distribution using the dose–volume histogram of the target volume [21]. The MLQ model instead of the LQ model was considered preferable because GRID therapy involves peak doses as high as 20 Gy, and consequently a significant volume of the tumour will receive doses greater than 10 Gy. In this high-dose range (>10 Gy) the LQ model tends to underestimate cell survival, as its radiosensitivities are obtained from the low-dose range experiments for characterising survival fraction [22,23,24]. A study demonstrated that the MLQ-based *EUD* is about 5% lower than that derived from Niemierko’s equation [25]. Because the MLQ model corrects the overkilling predicted by LQ model and Niemierko’s equation, we employed the *EUD* formulism proposed in Zhang et al.’s study, described in brief below [21] to obtain the *EUD* of GRID therapy.

The MLQ equation is as follows:

(4) $$SF_{i}=\exp\left(-\alpha \times D_{i}-\beta *G\left(\lambda \times T+\delta \times D_{i}\right)\times D_{i}^{2}\right)$$

*SF_i_* is the survival fraction at the dose *D_i_*_._ *α* and *β* are radiosensitivity parameters of the cell, $\;G\left(\lambda T\right)=\frac{2\left(\lambda T+e^{-\lambda T}-1\right)}{{\left(\lambda T\right)}^{2}}$, *λ* is the repair rate ($\lambda =\frac{ln2}{T_{1/2}}$), *T*_1/2_ is cell doubling time, *T*_1/2_= 1 h [26]; *δ* = 0.15 Gy^−1^ for both cancer and normal cells. *T* is the delivery time of the treatment (ranging from 4 to 7 min at 600 *MU*/min dose rate), assumed to be *T* = 5 min. The *SF* calculation is not very sensitive to *T*.

For cancer cells, we used a consensus value of α/β = 10 Gy. By assuming cancer cells as radiosensitive (SF(2Gy) = 0.3), semisensitive (SF(2Gy) = 0.5) and radioresistant (SF(2Gy) = 0.7), the individual α and β values of these three types of cancer cells can be derived from the LQ model. Therefore, for radiosensitive cancer cells, α = 0.502 Gy^−1^, and β = 0.0502 Gy^−2^; for semisensitive cancer cells, α = 0.289 Gy^−1^, and β = 0.0289 Gy^−2^; for radioresistant cancer cells, α = 0.149 Gy^−1^, and β = 0.0149 Gy^−2^, respectively. Similar assumptions can be made for normal tissue using α/β = 3 Gy. All radio-response parameters of the MLQ model are summarized in Table 1.

The average survival fraction $\bar{SF}$ was calculated using the following Equation (5):

(5) $$\bar{SF}=\frac{\sum_{1=1}^{i=N}SF_{i}\times f_{i}}{100}\;\overset{i=N}{\underset{i=1}{\sum }}f_{i}=100$$

*f_i_* is the fraction of target volume receiving dose *D_i_*. The average survival fraction was then utilized to solve the MLQ Equation (6) to determine the equivalent uniform dose (*EUD*) by solving the following equation for *EUD*:

(6) $$\exp(-\beta \times G\left(\lambda \times T+\delta \times \left(EUD\right)\right)\times {\left(EUD\right)}^{2}-\alpha \times \left(EUD\right)=\bar{SF}$$

For different prescription doses *D_p_*, we can calculate a list of corresponding *EUD*s. For a tumour located at 3 cm depth and treated with GRID therapy with different prescription doses, a 2nd order polynomial approximation equation is given in the following Equation (7):

(7) $$EUD=a_{0}+a_{1}\times D_{p}+a_{2}\times D_{p}^{2}$$

where, *a*_0_, *a*_1_ and *a*_2_ are the fitting coefficients.

We carried out a series of studies using a .decimal Inc.^TM^ GRID collimator and generated plans for different tumour sizes, shapes (either spherical or ellipsoidal), and depths in a flat phantom. The reference tables and dose–volume histogram (DVH) curves were derived based on recommendations of the “RSS GRID, Lattice, Microbeam and FLASH working group white paper” [27].

## 3. Results

### 3.1. Verification of TPS Dose Calculation Accuracy

**Figure 7:**
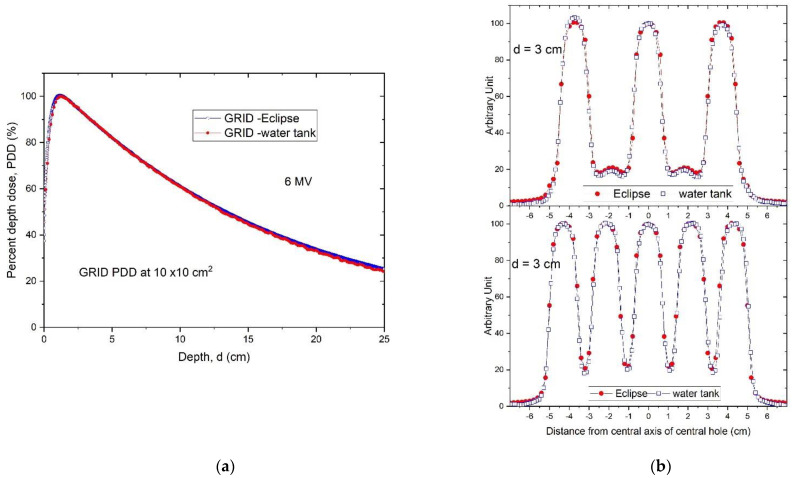
(**a**) Percent depth dose curves of a 10 × 10 cm^2^ GRID field. The Eclipse-calculated PDD (blue) was compared with the water tank-scanned data (red). (**b**) A comparison of radial (top) and transverse (bottom) dose profiles of GRID therapy at 3 cm depth calculated by Eclipse TPS (red) and measured by a water tank scanning system (blue).

The PDDs calculated using the TPS were compared to those measured with the water tank and were found to be in excellent agreement. This is illustrated in Figure 7a comparing PDD curves obtained by the TPS with the water tank scanning system. Similarly, high accuracy was observed in the dose profile comparison as illustrated in Figure 7b, in the comparison between the TPS-calculated and measured dose profiles at 3 cm depth.

### 3.2. Dosimetric Parameters of 3-D Tumour Targets in GRID Therapy

**Figure 8:**
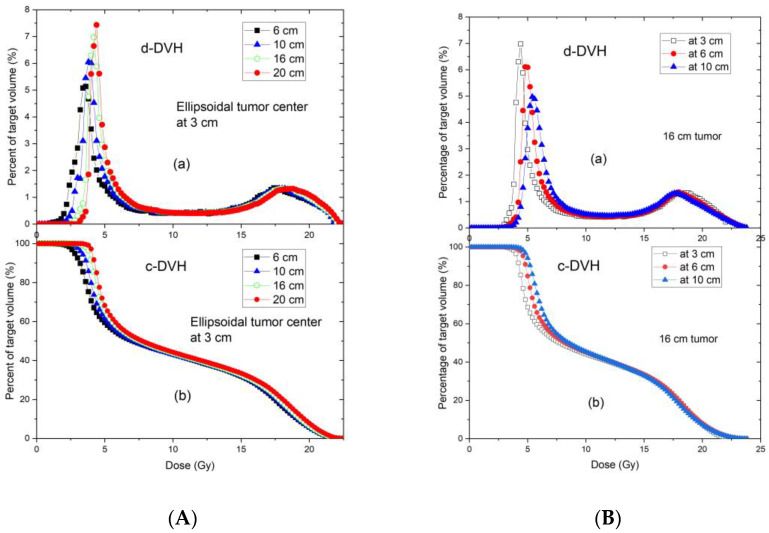
Differential (**a**) and cumulative (**b**) dose–volume histograms of tumors with different sizes centered at the depth of 3 cm (**A**), and the same size of tumor (16 cm) centered at 3, 6, and 10 depths (**B**). All tumors are ellipsoidal. The tumor height (along the beam direction) is 6 cm, in the plane perpendicular to the beam direction, the tumor shape is circular with the various diameters ranging from 6 to 20 cm.

Comparisons of TPS and measured data were assessed with respect to variations in tumour size and depth. Figure 8A,B show the DVH curves of tumours located at the same depth with different tumour sizes, and the same tumour size located at different depths, respectively. When the tumour size or depth increased, the TPS-calculated DVH curves shifted slightly towards higher doses.

Table 2 summarizes the doses covering 95% (D95), 90% (D90), 80% (D80), 70% (D70), 60% (D60), 50% (D50), 40% (D40), 30% (D30), 20% (D20), 10% (D10), and 5% (D5) of the target volume for different tumour sizes for a tumour depth of 3 cm (tumour center located at 3 cm depth). For a GRID prescription dose of 20 Gy, the standard deviations of all dose metrics for different tumour sizes were less than 0.5 Gy. Doses increased with increasing tumour size, and this increase was more noticeable in the lower dose metrics (D95, D90, and D80, where corresponding dose metric values are usually small) than in the higher dose metrics (D20, D10, and D5, where corresponding dose metric values are usually large).

Table 3 presents the same dose metrics as Table 2 for varying tumour center depths of 3, 6, and 10 cm and a constant tumour size of 16 cm. At the same prescription dose of 20 Gy, the standard deviations of the dose metrics at different depths were less than 0.6 Gy.

The DVH-derived dose tables were evaluated with respect to D10/D90, an important metric in SFRT. The respective peak-valley features calculated from the dose profiles and the D10/D90 and D5/D95 ratios are presented in Table 4 along with *PVDR*s. Table 4 shows that *PVDR* remains constant across varying tumour sizes, and decreases with increasing depth. Due to internal scattering, which will increase the dose in the shielded valley dose zones with increasing depth, *PVDR* decreased from 6.3 at 3 cm depth to 4.5 at 8 cm depth. Because a larger tumour tends to have a smaller ratio of D10/D90, mainly due to significant increase in D90 (the dose covering 90% of target volume), in the case of a 20 cm tumour centered at 8 cm depth, D10/D90 is found to be close to the *PVDR* (4.33 vs. 4.50).

In view of the importance of reporting *EUD*s in GRID therapy, the *EUD*s were calculated for different tumour scenarios. Table 5 summarizes the *EUD*s calculated using different radiosensitivities. The *EUD* of the radioresistant cancer cells was 30–40% higher than that of radiosensitive cancer cells for all tumour geometry scenarios.

Table 6 presents the *EUD*s calculated for the three types of normal tissue cells. Radioresistant normal tissue showed a greater *EUD* than radiosensitive normal tissue.

We calculated the *EUD*s of cancer cells at different prescription doses using a 16-cm tumour located at 3 cm depth as an example. Table 7 summarizes the *EUD*s of radiosensitive, semisensitive, and radioresistant cancer cells, respectively.

When calculating *EUD*s for different tumour sizes and averaging them at the same prescription dose, the approximate equations to estimate *EUD*s for different radio-sensitivities (*C*1, *C*2 and *C*3) at different prescription doses *D_p_* were best fitted by three 2nd order polynomial functions as follows:

(8) $$EUD_{C1}=0.7577+0.2447\times D_{p}-0.0011\times D_{p}^{2}\;\left(R^{2}=0.9998,\;D_{p}\geq 5\;Gy\right)$$

(9) $$EUD_{C2}=0.7315+0.3086\times D_{p}-0.0025\times D_{p}^{2}\;\left(R^{2}=0.9994,\;D_{p}\geq 5\;Gy\right)$$

(10) $$EUD_{C3}=0.4276+0.4219\times D_{p}-0.0048\times D_{p}^{2}\;\left(R^{2}=0.9994,\;\;D_{p}\geq 5\;Gy\right)$$

## 4. Discussion

Spatially fractionated radiation therapy is increasingly used in clinical practice. However, the clinical application of SFRT parameters that are profoundly different from familiar dosing concepts, and the variable capabilities of treatment planning systems to manage SFRT computations, present major challenges for clinical practitioners, particularly those who seek to newly establish an SFRT practice. Our results present proof of principle—based on our demonstration example of GRID collimator-based SFRT—that standardized reference tables to guide SFRT prescriptions can be developed, are robust, and thus may provide a practically applicable tool to assist GRID prescription and estimation of SFRT parameters.

The GRID collimator is an ideal scenario for the development and testing of these reference tables because it is a standard device that generates consistent dose heterogeneity properties. This also allows several dosimetric parameters to be directly derived from the proposed reference tables, as shown in our results.

Validation testing of our TPS-calculated dose profiles by water tank measurements showed a high accuracy with differences between TPS-calculated and measured GRID field doses within 3% at depths beyond the buildup region. Larger discrepancies were noted within the buildup region, as it is typically not accurately modeled by the TPS. Because the accuracy of small-field dose calculation is a longstanding concern [28], this overall result indicates that the Eclipse TPS accurately represents measured doses for this specific GRID collimator. While we cannot make a determination based on our results for other planning systems or other GRID collimator platforms, we expect that the results would likely be similar.

As we hypothesized, the commercially available GRID collimator showed consistent dose metrics for the range of studied treatment parameters. Regardless of tumour size, when located at the investigated 3 cm depth the standard deviations for the coverage doses of D95, D90, D80, … D20, D10, and D5 were less than 0.5 Gy (2.5% of prescription dose). Our results support that the proposed reference table data (Table 2) can be directly applied for treatment documentation for the treatment of tumours at a similar depth. For tumours located at greater depths (Table 3), the dose variation (standard deviation) is within 1 Gy, and we propose that in these cases the reference table can serve as a good estimate. When the information contained in Table 2 and Table 3 are considered together, it can be used to document additional tumour sizes and depths.

Our reference tables also provide a good estimate of the *PVDR*. *PVDR* is an important dosimetric parameter recommended in GRID therapy documentation as a measure of dose heterogeneity based on recent guidelines [27,29]. The determination and reporting of the dose heterogeneity is particularly important because preclinical data suggest that it is related to tumour response [30,31]. In 3D tumour volumes treated with GRID therapy, the computation and reporting of *PVDR* adds significant complexity to clinical practice. The peak/valley ratio varies inversely with depth, and it has been unclear at which particular depth to report the *PVDR* when characterising GRID therapy.

Our reference tables help address this challenge by providing DVH-based instead of single-depth-based *PVDR*. We believe a peak-valley dose metric determined from the 3D target volume’s DVH ratio of D10/D90 better reflects the dose heterogeneity of GRID therapy across the tumour volume than *PVDR* derived from the dose profile at a single depth. We provided D10/D90, along with D5/D95, and profile-based *PVDR* in a reference table (Table 4). This approach was based on the recommendation by the RSS Working Group’s white paper [27]. The D10/D90-based computation also prevents *PVDR*’s dependence from differences in the dose profiles resulting from being taken either in the inline or crossline direction. In addition, we favor D10/D90 over D5/D95 to describe the *PVDR* because D5 involves a volume which may be too small and D95 is located in a rapidly varying portion of the DVH. Therefore, although the D5 is closer to the prescription dose, we considered the D5/D95 metric less representative of the *PVDR*.

Our results support this view. The *PVDR* computations showed that for very large tumours (e.g., 20 cm in diameter and 16 cm in height centered at 8 cm depth), the D10/D90 and the peak/valley dose ratio from a dose profile at the tumour center are very similar, indicating that for large tumours the D10/D90 can represent the peak/valley ratio at the center.

The proposed reference tables include *EUD*, a concept that is highly relevant but particularly challenging for clinical physicists and clinicians. Our *EUD* results calculated for cancer cells of the following three different radiosensitivities are interesting: GRID therapy delivers different *EUD*s to different cells showing different radiosensitivities; we found that the *EUD* of radioresistant cancer cells was greater than the *EUD* of radiosensitive cancer cells (Table 5 and Table 6). This is explained by the more effective kill of radioresistant cancer cells in the high-dose regions. In contrast, for radiosensitive cells, these higher-dose regions are less “impactful” because radiosensitive cells are effectively killed with lower radiation doses. As a result, we calculated values for *EUD* of radioresistant cancer cells that were 30 to 40% greater than that of radiosensitive cells. This observation confirms that the treatment of radioresistant cancer cells will benefit relatively more from GRID therapy than that of radiosensitive tumour components [1,21]. In addition, our *EUD* results (Table 5 and Table 6) also indicate that the *EUD* increases with tumour size. This concept is well-supported by the notion that larger tumour volumes contain greater proportions of high-dose regions, resulting in increased *EUD*. Our observations therefore further support the use of GRID therapy for large, bulky tumours. The normal cells interspersed in the tumour volume will also be exposed to different *EUD*s since they, too have different radiosensitivities. Our results also indicate that the *EUD*s of normal tissue cells show a similar tendency to that of cancer cells. The *EUD*s of radioresistant normal tissue cells were 25 to 35% greater than those of radiosensitive normal cells. Comparing Table 5 with Table 6, the *EUD* of normal tissue is 3–7% smaller than the *EUD* of cancer cells, and this may imply less killing of normal tissues than the cancer cells in GRID therapy.

The need for better standardization of SFRT prescribing and reporting parameters has been well recognized [27]. These parameters include not only the prescription dose, but also the heterogeneity DVH metrics, *PVDR*, and *EUD* to model dose effects. While these unfamiliar dose heterogeneity parameters add significant complexity to clinical practice, they are indispensable for the progress of the field of SFRT [31,32], so that the clinical outcome results from clinical trials can be robustly compared and well-founded clinical practice recommendations can be developed.

Our developed reference tables can help facilitate and simplify the understanding of dose heterogeneity in the clinical environment, and thereby assist in the reporting requirements for GRID collimator-based SFRT. Based on our results that variations in dosimetric parameters are small, consistent, and predictable across different tumour sizes and depths, the use of the developed reference tables and graphs is clinically feasible. The tables and graphs can be used by radiation oncologists for selecting a treatment modality or for formulating a GRID treatment.

This capability may also aid clinical decision making on the choice of SFRT based on tumour-specific (volume, depth) parameters. It may further assist in the implementation of GRID therapy programs in institutions that newly implement this technique. 

Our tables may also serve to provide a reexamination of previously published GRID therapy clinical data, in which only tumour size, depth, and prescription dose information were reported. Such capability is of great importance for correlating clinical outcomes from previously treated patient cohorts with respect to specific dosimetric parameters that are postulated to relate to SFRT response [27]. This is particularly needed because of the current dearth of detailed dosimetric information linked to clinical local control and survival outcomes.

### Limitations and Caveats

For clinical use of the reference tables, however, users should be aware of their limitations. The current tables only apply to the .decimal Inc.^TM^ GRID collimator and a 6 MV beam. The reference tables (Table 2, Table 3, Table 5 and Table 6) presented here apply to a prescription dose of 20 Gy in 1 fraction, and conversion factors must be applied for other prescription doses as specified in the individual tables.

The reference tables apply to the homogenous tissue or water-equivalent material scenarios. For therapeutic photon beams corresponding to 6 MV or higher, bone does generally not result in significant heterogeneity, and the impact on *PVDR* is negligible. However, if lung tissue is involved, caution is advised, and the reference tables should not be used because of the possibility of introducing uncertainty.

Furthermore, DVH-derived dose parameters can be obtained from the reference tables for the tumour but not for organs at risk, because organs at risk may or may not be partially located within the GRID fields. We therefore encourage users to implement the GRID collimator in their TPS whenever possible to perform full 3D dose calculations corresponding to the patient’s individual anatomy.

## 5. Conclusions

This study developed practically applicable GRID dosimetry reference tables for use by physicists, dosimetrists, and physicians (applicable to a common commercially available standard GRID collimator) who face the common situation of lacking TPS availability for GRID planning. Observed variations in dosimetric parameters from different tumour sizes and depths are consistent and predictable. The resulting reference tables, graphs, and approximation equations developed in this study may serve as a guide for clinical decision-making on treatment selection, facilitate reporting requirements for GRID collimator-based SFRT in bulky tumours, and assist in the dose analysis of previously published GRID therapy clinical outcome data, which commonly lacks TPS-generated analysis of the heterogeneous tumour dose.

## Figures and Tables

**Table 1:** MLQ parameters of cancer cell lines (Radiosensitive C1, Semisensitive C2, and Radioresistant C3) and normal tissues (Radiosensitive N1, Semisensitive N2 and Radioresistant N3).

Cell Property	Cancer Cells			Normal Cells		
	C1	C2	C3	N1	N2	N3
α (Gy^−1^)	0.502	0.289	0.149	0.366	0.211	0.108
β (Gy^−2^)	0.0502	0.0289	0.0149	0.118	0.068	0.035
α/β (Gy)	10	10	10	3.0	3.0	3.0
T_1/2_ (h)	1	1	1	1	1	1
λ (h^−1^)	0.6931	0.6931	0.6931	0.6931	0.6931	0.6931
δ (Gy^−1^)	0.15	0.15	0.15	0.15	0.15	0.15

**Table 2:** Dose metrics (Gy) for different tumor sizes whose centers are located at 3 cm depth. The prescription dose of 20 Gy was given at the tumor center. Figure 5a describes the tumor geometry scenarios.

Metrics (Gy)	6 cm Sphere	8 cm Ellipsoid	10 cm Ellipsoid	12 cm Ellipsoid	16 cm Ellipsoid	20 cm Ellipsoid	Average (Gy)	Standard Deviation (Gy)
D95	2.82	3.30	3.31	3.65	3.85	4.10	3.51	0.46
D90	3.20	3.50	3.65	3.85	4.05	4.25	3.75	0.38
D80	3.61	3.85	4.00	4.15	4.35	4.55	4.09	0.34
D70	3.95	4.20	4.35	4.50	4.70	4.90	4.43	0.34
D60	5.00	5.02	5.20	5.30	5.60	5.70	5.30	0.29
D50	7.00	6.80	7.20	7.43	7.62	7.82	7.31	0.38
D40	11.61	10.82	11.50	11.50	11.80	12.10	11.56	0.43
D30	15.62	14.80	15.62	15.52	15.80	16.10	15.58	0.43
D20	17.52	17.00	17.60	17.60	17.82	18.05	17.60	0.35
D10	19.10	18.72	19.10	19.12	19.30	19.61	19.16	0.29
D5	20.20	19.80	20.10	20.10	20.30	20.50	20.17	0.23

Note: This table applies to a prescription dose of 20 Gy in 1 fraction. If the user’s prescription dose is other than 20 Gy, proportional adjustment factors must be employed. For example, for 15 Gy in 1 fraction, an adjustment factor of 0.75 (15 Gy/20 Gy = 0.75) is multiplied into the metrics. If the tumor center is off axis, the table may not be used.

**Table 3:** Dose metrics of a 16 cm diameter tumor located at different depths. The prescription dose of 20 Gy was given at the tumor center. Figure 5b describes the tumor geometry scenarios.

Metrics (Gy)	d = 3 cm	d = 6 cm	d = 10 cm	Average (Gy)	Standard Deviation (Gy)
D95	3.85	4.40	4.70	4.32	0.43
D90	4.05	4.65	5.02	4.57	0.49
D80	4.35	4.95	5.45	4.92	0.55
D70	4.70	5.35	5.90	5.32	0.60
D60	5.60	6.10	6.61	6.10	0.51
D50	7.62	8.00	8.30	7.97	0.34
D40	11.80	12.10	12.02	11.97	0.16
D30	15.80	15.90	15.65	15.78	0.13
D20	17.82	17.80	17.50	17.71	0.18
D10	19.30	19.42	19.21	19.31	0.11
D5	20.30	20.50	20.40	20.40	0.10

Note: This table applies to a prescription dose of 20 Gy in 1 fraction. If the user’s prescription dose is other than 20 Gy, proportional adjustment factors must be employed. For example, for 15 Gy in 1 fraction, an adjustment factor of 0.75 (15 Gy/20 Gy = 0.75) is multiplied into the metrics. If the tumor center is off axis, the table may not be used.

**Table 4:** D5/D95, D10/D90 and tumor center profile calculated peak/valley dose ratios (*PVDR*) of 3-D tumors treated by GRID therapy.

Tumor Dimension (cm)	D5/D95	D10/D90	*PVDR* Determined by the Profile at Tumor Center Depth
Tumor center at 3 cm depth with different tumor sizes			
6	7.16	5.97	6.33
8	6.00	5.35	6.33
10	6.07	5.23	6.33
12	5.51	4.97	6.33
16	5.27	4.77	6.33
20	5.64	4.61	6.33
average	5.94	5.15	6.33
Standard Deviation	0.67	0.49	0
16 cm tumor centered at different depths			
16 cm tumor at 3 cm	5.27	4.77	6.33
16 cm tumor at 6 cm	4.66	3.42	5.25
16 cm tumor at 10 cm	4.34	3.12	4.01
20 cm diameter tumor, 16 cm height			
20 cm tumor at 8 cm	5.18	4.33	4.50

Note: The referenced Table 4 is independent of the prescription dose. If the tumor center is off axis, the table may not be used.

**Table 5:** *EUD*s (Gy) for different tumor geometry scenarios with various radiosensitivities.

Tumor Dimension (cm)	*EUD* (Gy) Radiosensitive (C1)	*EUD* (Gy) Semisensitive (C2)	*EUD* (Gy) Radioresistant (C3)
Tumor center at 3 cm depth			
6	4.59	5.31	6.44
8	4.88	5.53	6.57
10	5.00	5.67	6.74
12	5.21	5.87	6.92
16	5.43	6.07	7.12
20	5.64	6.27	7.31
16 cm tumor at different depths			
16 cm tumor at 3 cm	5.43	6.07	7.12
16 cm tumor at 6 cm	5.99	6.62	7.63
16 cm tumor at 10 cm	6.39	7.02	7.99
20 cm tumor at 8 cm depth			
20 cm tumor at 8 cm	6.27	6.93	7.92

Note: This table applies to a prescription dose of 20 Gy in 1 fraction. If the user’s prescription dose is other than 20 Gy, proportional adjustment factors must be employed. For example, for 15 Gy in 1 fraction, an adjustment factor of 0.75 (15 Gy/20 Gy = 0.75) is multiplied into the metrics. If the tumor center is off axis, the table may not be used.

**Table 6:** *EUD*s (Gy) for 20 Gy prescription dose for interspersed normal cells with varying radio-sensitivities inside the tumors centered at 3, 6, and 8 cm depth. The tumor’s horizontal dimension ranges from 6 to 20 cm in diameter. If the tumor center is off axis, the table may not be used.

Tumor Dimension (cm)	*EUD* (Gy) Radiosensitive(N1)	*EUD* (Gy) Semisensitive (N2)	*EUD* (Gy) Radioresistant (N3)
Tumor center at 3 cm depth			
6	4.44	5.04	6.00
8	4.73	5.27	6.14
10	4.84	5.40	6.30
12	5.06	5.59	6.47
16	5.27	5.80	6.66
20	5.49	5.99	6.85
16 cm tumor at different depths			
16 cm tumor at 3 cm	5.27	5.80	6.66
16 cm tumor at 6 cm	5.82	6.34	7.17
16 cm tumor at 10 cm	6.21	6.73	7.54
20 cm tumor at 8 cm depth			
20 cm tumor at 8 cm	6.08	6.63	7.47

Note: This table applies to a prescription dose of 20 Gy in 1 fraction. If the user’s prescription dose is other than 20 Gy, proportional adjustment factors must be employed. For example, for 15 Gy in 1 fraction, an adjustment factor of 0.75 (15 Gy/20 Gy = 0.75) is multiplied into the metrics. If the tumor center is off axis, the table may not be used.

**Table 7:** *EUD*s (Gy) for cancer cells with different radiosensitivities at different prescription doses. A 16-cm diameter tumor centered at 3 cm depth was used as an example. If the tumor center is off axis, the table may not be used.

Prescription Dose (Gy)	*EUD* (Gy) C1 (Radiosensitive)	*EUD* (Gy) C2 (Semisensitive)	*EUD* (Gy) C3 (Radioresistant)
5	1.99	2.22	2.42
10	3.23	3.72	4.32
12	3.68	4.22	4.96
15	4.34	4.93	5.82
20	5.43	6.07	7.12
22	5.86	6.53	7.61
25	6.51	7.20	8.34

## Data Availability

The datasets used and/or analyzed during the current study are available from the corresponding author on reasonable request.

## References

[1] Mohiuddin M., Fujita M., Regine W.F., Megooni A.S., Ibbott G.S., Ahmed M.M. (1999). High-dose spatially-fractionated radiation (GRID): A new paradigm in the management of advanced cancers. Int. J. Radiat. Oncol. Biol. Phys..

[2] Mohiuddin M., Curtis D.L., Grizos W.T., Komarnicky L. (1990). Palliative treatment of advanced cancer using multiple nonconfluent pencil beam radiation. A pilot study. Cancer.

[3] Neuner G., Mohiuddin M.M., Vander Walde N., Goloubeva O., Ha J., Cedric X.Y., Regine W.F. (2012). High-dose spatially fractionated GRID radiation therapy (SFGRT): A comparison of treatment outcomes with Cerrobend vs. MLC SFGRT. Int. J. Radiat. Oncol. Biol. Phys..

[4] Penagaricano J., Phase I. (2017). Clinical Trial of GRID Therapy in Pediatric Osteosarcoma of the Extremity. https://clinicaltrials.gov/ct2/show/NCT03139318.

[5] Penagaricano J.A., Moros E.G., Ratanatharathorn V., Yan Y., Corry P. (2010). Evaluation of spatially fractionated radiotherapy (GRID) and definitive chemoradiotherapy with curative intent for locally advanced squamous cell carcinoma of the head and neck: Initial response rates and toxicity. Int. J. Radiat. Oncol. Biol. Phys..

[6] Mohiuddin M., Lynch C., Gao M., Hartsell W. (2020). Early clinical results of proton spatially fractionated GRID radiation therapy (SFGRT). Br. J. Radiol..

[7] Liberson F. (1933). The value of a multi-perforated screen in deep X-ray therapy. Radiology.

[8] Marks H. (1950). A new approach to the roentogen therapy of cancer with the use of a GRID. J. Mt. Sinai Hosp..

[9] Puri D.R., Chou W., Lee N. (2005). Intensity-modulated radiation therapy in head and neck cancers: Dosimetric advantages and update of clinical results. Am. J. Clin. Oncol..

[10] Marks H. (1952). Clinical experience with irradiation through a grid. Radiology.

[11] Miller R.C., Wilson K.G., Feola J.M., Urano M., Yaes R.J., McLaughlin P., Maruyama Y. (1992). Megavoltage grid total body irradiation of C3Hf/SED mice. Strahlenther. Onkol..

[12] Sathishkumar S., Dey S., Meigooni A.S., Regine W.F., Kudrimoti M.S., Ahmed M.M., Mohiuddin M. (2002). The impact of TNF-alpha induction on therapeutic efficacy following high dose spatially fractionated (GRID) radiation. Technol. Cancer Res. Treat..

[13] Sathishkumar S., Boyanovsky B., Karakashian A.A., Rozenova K., Giltiay N.V., Kudrimoti M., Mohiuddin M., Ahmed M.M., Nikolova-Karakashian M. (2005). Elevated sphingomyelinase activity and ceramide concentration in serum of patients undergoing high dose spatially fractionated radiation treatment: Implications for endothelial apoptosis. Cancer Biol. Ther..

[14] Huhn J.L., Regine W.F., Valentino J.P., Meigooni A.S., Kudrimoti M., Mohiuddin M. (2006). Spatially fractionated GRID radiation treatment of advanced neck disease associated with head and neck cancer. Technol. Cancer Res. Treat..

[15] Reiff J.E., Huq M.S., Mohiuddin M., Suntharalingam N. (1995). Dosimetric properties of megavoltage grid therapy. Int. J. Radiat. Oncol. Biol. Phys..

[16] Trapp J.V., Warrington A.P., Partridge M., Philps A., Glees J., Tait D., Ahmed R., Leach M.O., Webb S. (2004). Measurement of the three-dimensional distribution of radiation dose in grid therapy. Phys. Med. Biol..

[17] Meigooni A.S., Dou K., Meigooni N.J., Gnaster M., Awan S., Dini S., Johnson E.L. (2006). Dosimetric characteristics of a newly designed grid block for megavoltage photon radiation and its therapeutic advantage using a linear quadratic model. Med. Phys..

[18] Meigooni A.S., Gnaster M., Dou K., Johnson E.L., Meigooni N.J., Kudrimoti M. (2007). Dosimetric evaluation of parallel opposed spatially fractionated radiation therapy of deep-seated bulky tumors. Med. Phys..

[19] Howard M.E., Herman M.G., Grams M.P. (2020). Methodology for radiochromic film analysis using FilmQA Pro and ImageJ. PLoS ONE.

[20] Lewis D., Micke A., Yu X., Chan M.F. (2012). An efficient protocol for radiochromic film dosimetry combining calibration and measurement in a single scan. Med. Phys..

[21] Zhang H., Zhong H., Barth R.F., Cao M., Das I.J. (2014). Impact of dose size in single fraction spatially fractionated (grid) radiotherapy for melanoma. Med. Phys..

[22] Brenner D.J. (2008). The linear-quadratic model is an appropriate methodology for determining isoeffective doses at large doses per fraction. Semin. Radiat. Oncol..

[23] Chapman J.D., Gillespie C.J. (2012). The power of radiation biophysics-let’s use it. Int. J. Radiat. Oncol. Biol. Phys..

[24] Kirkpatrick J.P., Meyer J.J., Marks L.B. (2008). The linear-quadratic model is inappropriate to model high dose per fraction effects in radiosurgery. Semin. Radiat. Oncol..

[25] Saleh Y., Zhang H. (2017). Technical Note: Dosimetric impact of spherical applicator size in Intrabeam IORT for treating unicentric breast cancer lesions. Med. Phys..

[26] Guerrero M., Li X.A. (2003). Analysis of a large number of clinical studies for breast cancer radiotherapy: Estimation of radiobiological parameters for treatment planning. Phys. Med. Biol..

[27] Zhang H., Wu X., Zhang X., Chang S.X., Megooni A., Donnelly E.D., Ahmed M.M., Griffin R.J., Welsh J.S., Simone C.B. (2020). Photon GRID Radiation Therapy: A Physics and Dosimetry White Paper from the Radiosurgery Society (RSS) GRID/LATTICE, Microbeam and FLASH Radiotherapy Working Group. Radiat. Res..

[28] Lechner W., Primessnig A., Nenoff L., Wesolowska P., Izewska J., Georg D. (2020). The influence of errors in small field dosimetry on the dosimetric accuracy of treatment plans. Acta Oncol..

[29] Wu X., Perez N.C., Zheng Y., Li X., Jiang L., Amendola B.E., Xu B., Mayr N.A., Lu J.J., Hatoum G.F. (2020). The Technical and Clinical Implementation of LATTICE Radiation Therapy (LRT). Radiat. Res..

[30] Rivera J.N., Kierski T.M., Kasoji S.K., Abrantes A.S., Dayton P.A., Chang S.X. (2020). Conventional dose rate spatially-fractionated radiation therapy (SFRT) treatment response and its association with dosimetric parameters-A preclinical study in a Fischer 344 rat model. PLoS ONE.

[31] Griffin R.J., Ahmed M.M., Amendola B., Belyakov O., Bentzen S.M., Butterworth K.T., Chang S., Coleman C.N., Djonov V., Formenti S.C. (2020). Understanding High-Dose, Ultra-High Dose Rate, and Spatially Fractionated Radiation Therapy. Int. J. Radiat. Oncol. Biol. Phys..

[32] Billena C., Khan A.J. (2019). A Current Review of Spatial Fractionation: Back to the Future?. Int. J. Radiat Oncol. Biol. Phys..

